# Nurse’s life world in organ donation and tissue

**DOI:** 10.1590/0034-7167-2023-0521

**Published:** 2025-03-10

**Authors:** Karine de Melo Cezar Alves, Isabel Comassetto, Guilherme Oliveira de Albuquerque Malta, Regina Maria dos Santos, Gian Carlos Rodrigues do Nascimento, Igor Michel Ramos dos Santos

**Affiliations:** I Universidade Federal de Alagoas, Maceió, Alagoas, Brazil

**Keywords:** Tissue and Organ Procurement, Organ Transplantation, Nursing, Qualitative Research, Critical Care, Obtención de Tejidos y Órganos, Trasplante de Órganos, Enfermería, Investigación Cualitativa, Cuidados Críticos

## Abstract

**Objectives:**

to understand the experience of nurses immersed in the everyday world of organ and tissue donation.

**Methods:**

study on the social phenomenology of Alfred Schütz, carried out with 27 nurses who work on Intra-Hospital Committees for Donation of Organs and Tissues for Transplants in states in the Northeast of Brazil. Data were collected through phenomenological interviews and analyzed according to the adopted framework and compared with scientific productions.

**Results:**

the nurses’ experiences allowed us to glimpse the achievement and remaining immersed in the space of the world of daily life of organ and tissue donation.

**Final Considerations:**

the experience of nurses from different actions in the lives of people who depend on an organ and tissue transplant concerns the social relations established in the world of life.

## RESUMEN

**Objetivos:**

comprender la experiencia de enfermeras inmersas en el mundo cotidiano de la donación de órganos y tejidos.

**Métodos:**

estudio sobre la fenomenología social de Alfred Schütz, realizado con 27 enfermeros que actúan en Comités Intrahospitalarios de Donación de Órganos y Tejidos para Trasplantes en estados del Nordeste de Brasil. Los datos fueron recolectados a través de entrevistas fenomenológicas y analizados según el marco adoptado y comparados con producciones científicas.

**Resultados:**

las experiencias de las enfermeras permitieron vislumbrar el logro y la permanencia inmersa en el espacio del mundo de la vida cotidiana de la donación de órganos y tejidos.

**Consideraciones Finales:**

la experiencia de los enfermeros desde diferentes acciones en la vida de las personas que dependen de un trasplante de órganos y tejidos concierne a las relaciones sociales que se establecen en el mundo de la vida.

## INTRODUCTION

Organ and tissue donation and transplantation remain the most cost-effective and efficient therapy for individuals facing specific organ and tissue failure^(1)^. In 2021, 144,302 solid organ transplants were performed worldwide, with 38% being kidney transplants and 23% liver transplants. The United States, Spain, and France led these numbers, respectively^(2)^.

The United Kingdom stands out as an example of a nation that successfully doubled its organ donation rates over recent years. Similarly, Spain has maintained one of the highest donation rates globally, securing a position of international leadership in this context^(3)^. This achievement is attributed to distinctive features of Spain’s program, such as political and financial support and long-term commitment. Such success is sustained by a healthcare system with robust governmental structures closely tied to training programs, research, and campaigns encouraging organ and tissue donation^(4,5,6)^.

Currently, Brazil has 62,347 individuals waiting for transplant procedures, including people of both sexes and ranging in age from newborns to those over 65 years old, residing across all states in the country^(7)^. It is important to note that the global scenario continues to be influenced by the COVID-19 pandemic, which has posed challenges to resuming national organ and tissue donation and transplantation targets^(7,8)^.

Brazil ranks fourth globally in absolute numbers of kidney transplants and has an established and regulated transplant system; the public sector plays a crucial role through programs that have demonstrated progressive improvements in transplant procedures^(9)^. However, challenges remain, such as insufficient recent growth in donation rates, significant family refusal rates, considerable disparities between states and regions, financial limitations in some programs, and insufficient reporting of brain death cases^(10)^.

In this context, the process of organ and tissue donation involves various professionals, sectors, and stages, all of which each team member must follow carefully, whether directly or indirectly involved. These stages must comply with the legislation in each country and adhere to respective guidelines, clinical protocols, and care pathways to minimize the risk of adverse events during donation and transplantation^(11)^.

Understanding nurses’ experiences in organ and tissue donation is linked to a set of meanings, attitudes, phenomena, actions, and human relationships. According to social phenomenology’s theoretical and philosophical assumptions, we can analyze and understand human beings and their relations with the world^(12)^. As a research method, this framework offers a systematic path for investigating the experiences of nurses involved in organ and tissue donation while contextualizing their intersubjectivities in the social world^(13)^.

In social phenomenology, Alfred Schütz’s concept of the “life-world” refers to an intersubjective setting where individuals assume a social context—a biographical situation—and the entirety of their lived experiences. This term, used in the theoretical and methodological framework, guides this study, which explores the life-world of nurses in organ and tissue donation^(14)^.

When analyzing organ and tissue donation scenarios in the capital cities of Northeastern Brazil, questions arose regarding the number of effective donations each state carries out, as organ donation is closely tied to cultural issues. However, despite geographical and cultural proximity, we observed significant differences in the number of effective donations among certain states. Thus, this study hypothesizes the existence of a clarifying phenomenon behind the differing experiences of the nurses participating in the research.

## OBJECTIVES

To understand the experience of nurses immersed in the everyday life-world of organ and tissue donation.

## METHODS

### Ethical Aspects

This study adhered to all current ethical standards for research involving human participants. The Research Ethics Committee (CEP) reviewed and approved the research project. We informed all participants about the objectives, the research method used, and details regarding voluntary participation and the confidentiality of the information collected. Each participant voluntarily signed the Informed Consent Form (ICF). To ensure confidentiality and protect participants’ identities, we used alphanumeric codes.

### Theoretical-Methodological Framework

This study was based on the assumptions of Alfred Schütz’s social phenomenology. From this perspective, the existential motives that drive individuals to remain in the life-world are rooted in both the past and the future: “because motives” and “in-order-to motives”, respectively. In this study, we sought to understand the nurses’ experience within the life-world of organ donation. They live through a typical situation: the process of seeking organ and tissue donations. Thus, based on the “in-order-to” and “because motives”, we aimed to elucidate their experiences in the life-world^(14)^.

### Study Type

This is a qualitative study supported by Alfred Schütz’s social phenomenology. To ensure methodological rigor, we used the Consolidated Criteria for Reporting Qualitative Research (COREQ) as a tool to guide the research development.

### Study Setting

We conducted this study in five Intra-Hospital Commissions for Organ and Tissue Donation and Transplantation (CIHDOTT) located in five capitals of the Northeast region of Brazil (Recife, Salvador, Natal, Fortaleza, and Maceió). We noted significant quantitative differences in effective organ and tissue donations in the Northeast, with the state of Ceará leading, followed by Pernambuco, Bahia, Rio Grande do Norte, and Alagoas, respectively^(15)^.

### Data Source

The study included 27 nurses who had been working for at least one year in the CIHDOTTs of the capitals of Pernambuco, Bahia, Rio Grande do Norte, Ceará, and Alagoas. We interviewed three nurses from Maceió, nine from Fortaleza, six from Recife, three from Salvador, and six from Natal. Nurses on vacation or medical leave were excluded from the study.

### Data Collection and Organization

We contacted the participants after obtaining authorization from each research site’s management and nursing coordinators. Nurses who met the inclusion criteria were invited to participate, and interview dates, times, and locations were scheduled through phone contact facilitated by each nursing leadership. Thus, the researcher traveled to each city, staying for an average of three days to conduct the interviews.

Data collection occurred between June 2018 and February 2019, using phenomenological interviews. The interview began with an open-ended prompt: “Tell me about your experience as a nurse working with organ and tissue donation”. In addition, personal, socioeconomic, and professional information was gathered as part of the data collection protocol. All testimonies were included in the study, with no sample losses.

### Data Analysis

To support and provide consistency to the findings, we applied the theoretical-methodological framework of Alfred Schütz’s social phenomenology, which facilitates the analysis and understanding of individuals and their relationships with others^(14)^.

In this type of analysis, researchers first reach the state of *epoché*, which involves suspending all preconceptions to focus on the phenomenon. Following this, they apply sociological relevance rules—“What? Who? Where?”—to achieve a logical science that uses interviews to capture lived experiences. Based on this, the analysis focuses on the postulate of adequacy, which addresses action as adequately aligned with reality. This action is synthesized to generate logical-scientific rationality, following the framework established by Alfred Schütz^(13,14)^.

We fully transcribed all interviews. We then conducted multiple readings of each testimony to grasp the overall meaning. Afterward, they selected sections through prior categorization, dividing the text into units called “structures of subjective meanings”, which were repeatedly reviewed until the subjective meaning of the discourse was revealed.

## RESULTS

We analyzed social aspects and sociodemographic data to better understand each participant’s environment. Most participants were women, aged between 32 and 57, with experience in organ and tissue donation and related areas such as emergency care, intensive care, and surgery.

Using social phenomenology as the framework, our analysis of the interviews uncovered the hidden phenomenon in the experiences of nurses involved in the life-world of organ and tissue donation. The categories identified are described below.

### The Motivation of Nurses to Enter and Remain Immersed in the Life-World of Organ and Tissue Donation

Understanding the action phenomenon involves interpreting the existential motives, which are the “in-order-to motives” related to the future of the nurses in the study. The “because motives” are linked to their accumulated knowledge and past experiences.

*We hear such stories here that leave us perplexed. You do it because you intend to help someone down the line, someone I don’t even know, but we know it will help someone. That’s why we stay here, searching for donors*. (P3)

After recognizing the existential motives of nurses immersed in the life-world of organ and tissue donation, their experience is profoundly marked by the social action of implementing and executing the brain death protocol.

### Nurses’ Approach and Integration into the Life-World of Organ and Tissue Donation

Daily and professional experiences bring nurses closer to the life-world of organ and tissue donation. This enriches their scientific knowledge, expanding the specific framework, which continues to transform throughout their concrete existence. Alfred Schütz refers to this involvement as the “biographical situation of Being”, which emerged as the second concrete category of lived experience in this study.

Schütz’s concept of the “social Being” shows that the nurse, as a social Being, lives in an everyday world based on a life purpose shaped by previous experiences. These experiences act as a reference code, serving as guidance and manifesting as a form of knowledge. Nurses who have witnessed the suffering of people waiting for organs to survive are drawn closer to the life-world of donation:

*I have extensive ICU experience. That’s where I got closer to the donation process. When there were patients with brain death, I already had a different perspective on those types of patients—so much so that my graduation thesis was on brain death, and that’s where my passion started.* (P9)

### Nurses’ Engagement with the Life-World of Organ and Tissue Donation

In the context of organ and tissue donation, nursing care is carried out with complexity and is considered by society to be of great importance, becoming a social action. From Alfred Schütz’s perspective, the nurse is the protagonist of the life-world.

*Today, we conduct a systematic active search in the hospital: I go to the patient’s bedside, check if they’re on mechanical ventilation, evaluate their Glasgow scale, check for respiratory drive, examine pupils and reflexes, and then monitor these patients daily. If any signs arise for a potential brain death protocol opening, we evaluate the test results, issue the donor alert, and confirm the sedation half-life and temperature before starting the protocol.* (P25)

In the life-world of the nurses in this study, family-centered care is the gold standard in nursing during the organ and tissue donation process.

*I think it’s a matter of listening and understanding because sometimes they don’t understand, and you’ve got to see things from their perspective. You have to stop, look, and listen before you can begin explaining the whole process to them, and then you win them over. That’s magical!* (P19)

The culmination of the story constructed by CIHDOTT nurses, as they navigate the life-world of organ and tissue donation, occurs when they delve deeply into Being amidst the loss. These professionals embrace the families, comfort their pain, and, with great intimacy, guide them toward the possibility of organ donation.

*This contact with the family is wonderful. For me, it’s the best part, but it’s also the hardest. It’s the part that still gives me butterflies every time, makes my mouth go dry, and my heart pound. And it’s not because I don’t know what to do—it’s because it’s such a delicate moment. It’s a moment when we have to tread lightly: every word spoken out of context ruins everything; a gesture, non-verbal posture, can destroy everything.* (P18)

## DISCUSSION

Nurses immersed in the life-world of organ and tissue donation are driven by a profound goal: saving lives. They understand the significance of their role, even though they may never meet those who will benefit from a potential transplant. This objective provides motivation and meaning to their existence as nurse-beings, placing them in a strategic position to identify potential donors due to their inherent caregiving nature^(16,17,18)^.

The “because motives” evident in the lived experiences of these nurses highlight the duality of emotions they face: the sadness of loss and the joy of donation^(19)^. In the context of organ and tissue donation, nursing care is described as critical care, characterized by complexity and the technological environment in which it is carried out, whether in intensive care units or surgical centers; this type of care requires a solid knowledge base, dynamism, and efficiency from the nurse^(18,20)^.

Being part of the organ and tissue donation process carries deep meaning, transcending the mere technical execution of protocols. It is interwoven with the societal connotations that organ and tissue transplantation represents, especially about the ethical tensions arising in end-of-life care. For nurses, this becomes a delicate and challenging professional task that requires balancing the delivery of end-of-life care with the preservation of organs for donation^(21,22)^.

Insufficient knowledge about this topic is one of the factors behind the scarcity and fragility of the educational approach to preparing nurses for organ and tissue donation in undergraduate and postgraduate health programs in Brazil. Nurses are not adequately trained to identify potential donors or care for neurocritical patients who will soon become organ and tissue donors, which can ultimately affect transplant outcomes^(8,23,24,25)^.

CIHDOTT nurses are increasingly gaining recognition, building solid relationships with multidisciplinary teams, and establishing ties with various institutions^(26,27)^. They are acknowledged as experts in caring for potential donors and their families, holding the responsibility of managing the steps involved in organ and tissue donation, including active searches, identification, evaluation, validation, donor notification, family interviews, coordination of the operating room, and document submission^(26,28)^.

Effective communication between CIHDOTT teams and care teams is critical for the success of the organ and tissue donation process. This communication is a skill developed by health teams that achieve excellence in care quality and is a technique employed by multidisciplinary teams within their scope of practice^(26,28)^.

When intensive care nurses are involved in the process, families often emphasize the importance of their close relationship with the nurses in coping with the death of a loved one. Families highlight these professionals’ kindness, compassion, and attentiveness, recognizing that nursing care plays a pivotal role in the decision to donate^(29,30)^.

Once a bond is established, with the family’s active involvement in the brain death protocol, there is often an affirmative option for organ and tissue donation. Establishing a supportive relationship with the family serves as a humanization strategy within the brain death protocol, offering resources and reducing the family’s anxiety throughout the process^(31)^.

The success of any donation depends on the early detection of potential donors and their prompt referral to the appropriate professionals for evaluation and conversion into actual donors^(32)^. Failures at this protocol stage reflect poor quality indicators in the organ donation process, often caused by technical inadequacies or professional negligence^(33)^.

The role of professionals involved in organ and tissue donor procurement and the maintenance of deceased potential donors goes beyond managing hemodynamic aspects. It also involves supporting the family and delivering difficult news^(34)^. Family support and interviews are the most challenging moments due to the pain and suffering that death represents. At this stage, professionals must delicately convey the finality of death, taking away the hope of recovery for a loved one and confronting the reality of their passing.

### Study limitations

Since this is a qualitative study, the presented results reflect specific characteristics of the nurses studied. The reality depicted may differ from other contexts, which limits the generalization of the findings. Thus, further investigative possibilities need to be explored.

### Contributions to the Field

By understanding the diverse experiences of professionals from different regions of the country who face varied realities regarding the donation landscape, we believe these findings will help facilitate nursing practices in this area of healthcare. The study can offer valuable support in guiding care and teaching while also helping direct the implementation of new public policies aimed at organ and tissue donation. This could bring hope to those waiting for transplants.

## FINAL CONSIDERATIONS

Living immersed in the life-world of organ and tissue donation is filled with challenges that nurses overcome throughout their professional journey. Even before entering this field, they prepare to work in this cause, accumulating lived experience and scientific knowledge to handle the brain death process and organ and tissue donation. Being a nurse in this process means providing high-quality care across all dimensions impacted by organ donation and transplantation, whether directly or indirectly. It involves caring for all those engaged in the process, including families, potential donors, and possible organ recipients, offering society ethical, dignified, and humanistic nursing care.
