# Supplementary material for: Nurse’s life world in organ donation and tissue
Source: Rev Bras Enferm. 2025 Mar 10;78(1):e20230521. doi: 10.1590/0034-7167-2023-0521 (PMC11893057; doi:10.1590/0034-7167-2023-0521)
Supplement: Translation

# Translation: Mundo da vida do enfermeiro na doação de órgãos e tecidos

**DOI:** 10.1590/0034-7167-2023-0521pt

**Authors:** Karine de Melo Cezar Alves, Isabel Comassetto, Guilherme Oliveira de Albuquerque Malta, Regina Maria dos Santos, Gian Carlos Rodrigues do Nascimento, Igor Michel Ramos dos Santos

**Affiliations:** I Universidade Federal de Alagoas, Maceió, Alagoas, Brasil

**Keywords:** Obtenção de Tecidos e Órgãos, Transplante, Enfermagem, Pesquisa Qualitativa, Cuidados Críticos

## Abstract

**Objetivos:**

compreender a experiência dos enfermeiros imersos no mundo da vida cotidiana da doação de órgãos e tecidos.

**Métodos:**

este estudo foi fundamentado em pressupostos da fenomenologia social de Alfred Schütz e realizado com 27 enfermeiros que atuam nas Comissões Intra-Hospitalares de Doação de Órgãos e Tecidos para Transplantes em estados do Nordeste do Brasil. Os dados foram coletados por entrevista fenomenológica, analisados conforme referencial adotado e confrontados com produções científicas.

**Resultados:**

as experiências dos enfermeiros permitiram vislumbrar a conquista e o permanecimento imerso no espaço do mundo da vida cotidiana da doação de órgãos e tecidos.

**Considerações Finais:**

a experiência dos enfermeiros advinda de ações diferenciadas na vida das pessoas que dependem de um transplante de órgãos e tecidos liga-se às relações sociais instituídas no mundo da vida.

## INTRODUÇÃO

A doação e o transplante de órgãos permanecem como a terapêutica mais econômica e eficaz para indivíduos que enfrentam a insuficiência de órgãos e tecidos específicos.^(1)^ Em 2021, foram realizados 144.302 transplantes de órgãos sólidos no mundo, dos quais 38% foram renais e 23% hepáticos, com os Estados Unidos, Espanha e França liderando o ranking, respectivamente^(2)^.

O Reino Unido sobressai-se como exemplo de nação que conseguiu duplicar suas taxas de doações de órgãos ao longo dos últimos anos. Do mesmo modo, a Espanha tem se destacado por manter uma das taxas de doação mais elevadas do mundo, assumindo uma posição de liderança global nesse contexto^(3)^. Tal sucesso pode ser atribuído aos traços distintivos do programa espanhol, como o apoio político e financeiro e o comprometimento de longo prazo. Trata-se de uma conquista sustentada por um sistema de saúde com estruturas governamentais sólidas, inclusivas e intimamente ligadas a programas voltados para treinamentos, pesquisas e campanhas que incentivam doações de órgãos e tecidos^(4,5,6)^.

Atualmente, o Brasil registra um total de 62.347 indivíduos aguardando por procedimentos de transplante. Essa lista engloba pessoas de ambos os sexos, com idades variando de 0 a mais de 65 anos, residentes em todos os estados do país^(7)^. É válido ressaltar que o cenário global ainda é influenciado pela pandemia de COVID-19, o que tem acarretado desafios na retomada das metas concernentes a doações e transplantes no território nacional^(7,8)^.

O Brasil ocupa a quar ta posição em termos absolutos de transplantes renais realizados globalmente e mantém um sistema de transplantes estabelecido e regulamentado; o setor público desempenha um papel essencial por meio de programas, cujos resultados têm demonstrado progressiva melhora no âmbito dos procedimentos de transplantes^(9)^. Contudo, persistem desafios a serem superados, tais como crescimento recente insuficiente nas taxas de doação, índices consideráveis de recusa familiar, disparidades significativas entre estados e regiões, limitações financeiras em alguns programas e notificação ainda insuficiente de morte encefálica^(10)^.

Nesse cenário, o processo de doação de órgãos e tecidos envolve profissionais, setores e etapas distintas, as quais devem ser seguidas cuidadosamente por cada membro da equipe que nele atua, seja de forma direta, seja indireta. As etapas devem ser cumpridas considerando a legislação vigente em cada país, segundo as respectivas diretrizes, guias clínicos e protocolos assistenciais, que possibilitam minimizar o risco de eventos adversos na doação e transplante^(11)^.

A compreensão da experiência dos enfermeiros na doação de órgãos e tecidos está ligada a um conjunto de significados, atitudes, fenômenos, ações e relações humanas. Consoante os pressupostos teóricos e filosóficos da fenomenologia social, é possível analisar e compreender o homem e suas relações com o mundo^(12)^. Como método de pesquisa, é sugerido um caminho sistemático para investigar a experiência dos enfermeiros envolvidos na doação de órgãos e tecidos, além de contextualizar suas intersubjetividades no mundo social^(13)^.

Assim, no contexto da fenomenologia social, o conceito de “mundo da vida” dado por Alfred Schütz refere-se a um cenário intersubjetivo em que o ser humano assume um contexto social — situação biográfica —, assim como a totalidade de experiências vividas. É um termo utilizado pelo referencial teórico e metodológico, que, neste estudo, abordou o mundo da vida dos enfermeiros na doação de órgãos e tecidos^(14)^.

Ao analisar cenários de doação de órgãos e tecidos no âmbito das capitais da Região Nordeste, surgiram questionamentos sobre os números de doações efetivas que cada estado desenvolvia, já que a doação de órgãos está muito associada a questões culturais. Porém, apesar da proximidade geográfica e cultural, observaram-se diferenças significativas no número de doações efetivas de determinados estados. Sendo assim, este estudo traz a hipótese da existência de um fenômeno esclarecedor por trás das diferentes experiências dos enfermeiros participantes da pesquisa.

## OBJETIVOS

Compreender a experiência dos enfermeiros imersos no mundo da vida cotidiana da doação de órgãos e tecidos.

## MÉTODOS

### Aspectos Éticos

Esta pesquisa atendeu a todos os preceitos éticos vigentes para pesquisas que envolvem seres humanos. O projeto foi apreciado pelo Comitê de Ética e Pesquisa (CEP), que emitiu parecer favorável à pesquisa. Todos os participantes foram esclarecidos quanto aos objetivos, método utilizado na pesquisa e informações sobre a participação voluntária e sigilo acerca das informações coletadas. Todos assinaram espontaneamente o Termo de Consentimento Livre e Esclarecido (TCLE). Para garantir o sigilo das informações coletadas e a preservação da identidade dos participantes, foram utilizados códigos alfanuméricos.

### Referencial Teórico-Metodológico

Este estudo fundamentou-se em pressupostos da fenomenologia social de Alfred Schütz. Sob essa perspectiva, os motivos existenciais que fazem o ser humano permanecer no mundo da vida são baseados no passado e futuro: os “motivos porque” e “motivos para”, respectivamente. Neste estudo, buscou-se compreender a experiência dos enfermeiros no mundo da vida da doação de órgãos. Eles vivem uma situação típica: a realização da busca pela doação de órgãos e tecidos. Assim, com base nos “motivos para” e “motivos porque”, buscou-se elucidar sua experiência no mundo da vida^(14)^.

### Tipo de estudo

Trata-se de um estudo qualitativo, apoiado na fenomenologia social de Alfred Schütz. Para manter o rigor metodológico, foi utilizado o protocolo *Consolidated Criteria for Reporting Qualitative Research* (COREQ) como ferramenta de apoio para o desenvolvimento da pesquisa.

### Cenário do Estudo

A pesquisa foi realizada em cinco Comissões Intra-Hospitalares de Doação de Órgãos e Tecidos para Transplante (CIHDOTT), localizadas em cinco capitais da Região Nordeste do país (Recife, Salvador, Natal, Fortaleza e Maceió). Foram significativas as diferenças quantitativas nas doações de órgãos e tecidos efetivas no Nordeste, lideradas pelo estado do Ceará, seguido por Pernambuco, Bahia, Rio Grande do Norte e Alagoas, respectivamente^(15)^.

### Fonte de Dados

Participaram do estudo 27 enfermeiros atuantes, há pelo menos um ano, nas CIHDOTTs das capitais dos estados de Pernambuco, Bahia, Rio Grande do Norte, Ceará e Alagoas. Foram entrevistados três enfermeiros de Maceió, nove de Fortaleza, seis de Recife, três de Salvador e seis de Natal. Foram excluídos os que estavam em férias e/ou sob atestado médico.

### Coleta e organização dos dados

O contato com os participantes deste estudo foi realizado após autorização da gestão e coordenadora de enfermagem de cada local de pesquisa. Os enfermeiros que atendiam aos critérios de inclusão da pesquisa foram convidados a participar, sendo agendados data, horário e local de realização da entrevista, após contato telefônico por intermédio de cada liderança de enfermagem. Assim, a pesquisadora programou a viagem até cada cidade e permaneceu, em média, por três dias para a realização das entrevistas.

A coleta de depoimentos foi realizada entre junho de 2018 e fevereiro de 2019, utilizando-se como instrumento a entrevista fenomenológica. Ao iniciar a entrevista, foi posta a seguinte questão disparadora para uma entrevista aberta: “Conte sobre sua experiência como enfermeiro que trabalha com doação de órgãos e tecidos”. Além disso, no roteiro de coleta de dados, foram acrescentadas informações pessoais, socioeconômicas e profissionais. Todos os depoimentos foram incluídos no estudo, não havendo perdas amostrais.

### Análise dos dados

Para subsidiar e trazer consistência aos resultados encontrados, fez-se uso do referencial teórico-metodológico sob a perspectiva de Alfred Schütz, que permite a análise e compreensão do homem e relações de ser com o outro^(14)^.

Nesse tipo de análise, inicialmente o pesquisador atinge o estado de *epoché*, que é a suspensão de todos os pré-julgamentos, direcionando o olhar para o fenômeno. Em seguida, ele adota as regras de relevância sociológica, “O quê? Quem? Onde?”, para alcançar uma ciência lógica, a qual utiliza a entrevista como instrumento de obtenção do vivido. Com base nisso, concentra-se no postulado da adequação, que trata da ação compreendida como adequada à realidade; tal ação é sintetizada gerando a racionalidade lógico-científica enquanto resultado da análise baseada em Alfred Schütz^(13,14)^.

As entrevistas foram transcritas na íntegra. Em seguida, foram realizadas diversas leituras de cada um dos discursos das experiências com o propósito de apreender o sentido do todo. Logo depois, foram selecionadas por meio de categorização prévia, dividindo o texto em unidades chamadas “estruturas de significados subjetivos”, as quais foram lidas repetidas vezes até desvelarem o significado subjetivo do discurso.

## RESULTADOS

Para compreender melhor o ambiente em que cada participante estava inserido, foram analisados os aspectos sociais e os dados sociodemográficos, sendo a grande maioria do sexo feminino, com faixa etária entre 32 e 57 anos, experiência em doação de órgãos e tecidos e áreas afins como urgência e emergência, terapia intensiva e cirurgia.

A análise das entrevistas, por meio da fenomenologia social, revelou o fenômeno oculto nas experiências relatadas pelos enfermeiros envolvidos no mundo da vida na doação de órgãos e tecidos. A seguir, estão descritas as categorias.

### A motivação do enfermeiro para adentrar e permanecer imerso no mundo da vida na doação de órgãos e tecidos

A compreensão do fenômeno da ação perpassa pela interpretação dos motivos existenciais, os quais são os “motivos para” que estão relacionados com o futuro dos enfermeiros participantes do estudo. Já os “motivos porque” estão atrelados ao acervo de conhecimentos e experiências vividas por ele.

*A gente escuta cada história aqui que ficamos perplexos. Você faz porque você tem a intenção de ajudar alguém lá na ponta, que eu não sei nem quem é, mas a gente sabe que ajudará alguém. É nesse intuito que a gente fica aqui procurando doador*. (P3)

Após reconhecer os motivos existenciais do enfermeiro imerso no mundo da vida na doação de órgãos e tecidos, sua vivência é marcada efetivamente pela ação social da implementação e efetivação do protocolo de morte encefálica.

### A aproximação e a conquista do espaço do enfermeiro no mundo da vida na doação de órgãos e tecidos

As experiências do cotidiano e da vida profissional levam os enfermeiros a aproximarem-se do mundo da vida na doação de órgãos e tecidos. Com isso, enriquecem os conhecimentos científicos, ampliando o arcabouço específico, que continua em transformação durante toda a sua existência concreta. Alfred Schütz chama esse envolvimento de “Situação biográfica do Ser”, que, neste estudo, emergiu como a segunda categoria concreta dos vividos.

A situação biográfica do “Ser social” descrita por Alfred Schütz mostra aqui que o enfermeiro é um “Ser social” vivendo em um mundo cotidiano baseado em um propósito de vida, fundamentado em trajetórias com experiências anteriores. Essas experiências funcionam como um código de referência, servindo como orientação e aparecendo sob uma forma de conhecimento. São profissionais que já presenciaram o sofrimento de pessoas que precisam de um órgão para sobreviver, e isso aproxima o Ser ao mundo da vida na doação de órgãos:

*Tenho uma experiência extensa em UTI. Foi aí que eu me aproximei do processo de doação. Quando tinha pacientes com morte encefálica, eu já tinha aquele olhar diferenciado para esse tipo de paciente. Tanto que a monografia da minha graduação foi em morte encefálica, e daí começou a minha paixão.* (P9)

### O viver do enfermeiro para o mundo da vida na doação de órgãos e tecidos

No contexto da doação de órgãos e tecidos, o cuidado de enfermagem é exercido com complexidade. Ele é considerado pela sociedade como um trabalho de grande importância, tornando-se uma ação social. Sob a ótica de Alfred Schütz, o enfermeiro é protagonista do mundo da vida.

*Hoje fazemos uma busca ativa sistematizada no hospital: vou no leito do paciente, olho se ele está com ventilação mecânica, avalio através da escala de Glasgow, verifico se tem driver respiratório, vejo pupilas, reflexos, e aí a gente vai acompanhando diariamente esses pacientes. Caso surja algum sinal para uma possível abertura de protocolo de morte encefálica, aí avaliamos os resultados de exame, abrimos o alerta doador e confirmamos o tempo de meiavida de sedação e temperatura para poder abrir o protocolo.* (P25)

No mundo da vida dos enfermeiros participantes do estudo, o cuidado centrado na família é padrão-ouro para os atendimentos de enfermagem no processo de doação de órgãos e tecidos.

*Acho que é uma questão de escutar e entender, porque eles às vezes não entendem, não compreendem, e você tem que entender o lado deles; você tem que parar e olhar e escutar para depois você começar a explicar todo o processo para eles e aí você os ganha. Isso é mágico!* (P19)

O desfecho da história construída pelos enfermeiros das CIHDOTTs, por todo o caminhar do mundo vida da doação de órgãos e tecidos, realiza-se sob os motivos existenciais traçados quando eles se inserem com profundidade no Ser diante da perda. Então esses profissionais acolhem-no, confortam sua dor e, com muita intimidade, iluminam-no para a possibilidade de doação de órgãos.

*Então, esse contato com a família é uma coisa maravilhosa. Pra mim, é a melhor parte, é a mais difícil. É a que eu sinto um arrepio na barriga até hoje toda vez, a boca seca, o coração batendo forte. E não é porque eu não saiba fazer, é porque é um momento muito delicado. É um momento em que a gente tem que ir nas pontas dos dedos: cada palavra dita fora do contexto estraga tudo; um gesto, uma postura não verbal estraga tudo.* (P18)

## DISCUSSÃO

A atuação do enfermeiro imerso no mundo da vida da doação de órgãos e tecidos é projetada no intuito de salvar vidas. Ele reconhece a importância das suas práticas nesse mundo, apesar de não conhecer aqueles que serão beneficiados com um possível transplante. Tal objetivo o motiva e dá significado à sua existência enquanto Ser-enfermeiro. Assim, esse profissional ocupa uma posição estratégica para identificar os potenciais doadores, por sua natureza de exercer o cuidado^(16,17,18)^.

Os “motivos porque” no vivido dos enfermeiros imersos no mundo da vida na doação de órgãos e tecidos evidenciam-se na dualidade de sentimentos diante da tristeza da perda e alegria da doação^(19)^.

No contexto de doação de órgãos e tecidos, os cuidados da enfermagem são descritos como cuidados críticos, que são complexos e executados em um ambiente tecnologicamente rico, seja em unidades de terapias intensivas, seja em centro cirúrgicos, exigindo uma forte base de conhecimento, dinamismo e eficiência por parte do profissional^(18,20)^.

Fazer parte do processo de doação de órgãos e tecidos é uma função permeada de significados, que vai além da execução técnica de um protocolo. Liga-se com a conotação que o transplante de órgãos e tecidos carrega diante da sociedade, destacando-se as tensões éticas que tratam os cuidados de fim de vida como um procedimento. Esse processo torna-se um grande desafio profissional, definido pelo enfermeiro como delicado, de difícil execução e que exige um compromisso difícil entre os cuidados de fim de vida e a preservação de órgãos^(21,22)^.

O conhecimento insuficiente sobre essa temática está por trás da escassez e fragilidade da abordagem voltada ao preparo do enfermeiro para a doação de órgãos e tecidos nas grades curriculares dos cursos de graduação e pós-graduação em saúde do país. O profissional não é treinado para identificar potenciais doadores e cuidar de pacientes neurocríticos que serão em breve doadores de órgãos e tecidos, o que afeta o resultado dos transplantes efetivos^(8,23,24,25)^.

O enfermeiro da CIHDOTT vem adquirindo novos espaços, conquistando as equipes assistentes e, assim, consolidando vínculos com as mais diversas instituições^(26,27)^. É reconhecido como referência em cuidados ao potencial doador e sua família. Tem a responsabilidade e compromisso de gerenciar as etapas do processo de doação de órgãos e tecidos, como a busca ativa, identificação, avaliação, validação, notificação do potencial doador, entrevista com a família, coordenação da sala cirúrgica e envio de documentos^(26,27,28)^.

A comunicação entre as equipes da CIHDOTT e as equipes assistenciais é essencial para a execução do processo de doação de órgãos e tecidos. É uma habilidade desenvolvida nas equipes de saúde que alcançam níveis de excelência na qualidade da assistência e uma técnica aplicada pelas equipes multidisciplinares em seu âmbito de atuação^(26,27,28)^.

Quando o enfermeiro intensivista faz parte do processo, as famílias enfatizam como o relacionamento próximo com eles é fundamental para o enfrentamento da morte do familiar: destacam a gentileza, carinho e atenção desse profissional, mostrando que o cuidado de enfermagem é considerado influente na decisão para a doação^(29,30)^.

A partir do momento que o profissional fortalece vínculo, com a participação ativa da família dentro do protocolo de morte encefálica, existe uma afirmativa/opção ao final para a doação de órgãos e tecidos. Estabelecer uma relação de ajuda com os familiares funciona como uma estratégia de humanização do protocolo de morte encefálica, de modo que oferece recursos e diminui a ansiedade da família no percorrer do processo^(31)^.

O sucesso de qualquer doação requer que potenciais doadores sejam detectados e encaminhados precocemente aos profissionais responsáveis para sua avaliação e conversão em doadores reais^(32)^. Falhas nessa etapa do protocolo repercutem como um indicador de qualidade frágil diante da necessidade de efetivação de doação de órgãos, o que pode ocorrer por despreparo técnico e/ou negligência do profissional^(33)^.

A atuação dos profissionais que trabalham com procura de órgãos e tecidos e manutenção do potencial doador falecido não se restringe aos aspectos hemodinâmicos, mas inclui também o acolhimento familiar e a comunicação de más notícias^(34)^. O acolhimento e a entrevista familiar são os momentos mais desafiadores, devido ao cenário de dor e sofrimento que a morte representa, em que o profissional retira as esperanças de uma possível melhora daquele ente querido e traz a realidade da finitude.

### Limitações do estudo

Por ser um estudo qualitativo, os resultados apresentados constituem evidências que traduzem características específicas dos enfermeiros estudados. A realidade retratada pode diferenciar-se de outra, o que impede a generalização dos resultados. Por isso, outras possibilidades investigativas precisam ser implementadas.

### Contribuições para Área

De posse do conhecimento das várias experiências vividas por profissionais que habitam uma mesma região do país e que possuem realidades diversas quanto ao cenário das doações, acredita-se que os achados poderão contribuir para facilitar as práticas assistenciais nesse campo da saúde, proporcionando o suporte ideal para a orientação na assistência e no ensino. Além disso, poderão nortear uma necessária implantação de novas políticas públicas voltadas à doação de órgãos e tecidos, a fim de promover esperança de vida às pessoas que estão na fila de espera.

## CONSIDERAÇÕES FINAIS

Viver imerso no mundo da doação de órgãos e tecidos é permeado por desafios que os enfermeiros superam ao longo de sua trajetória. Desde antes de ingressarem nesse campo, já se preparam para atuar nessa causa, acumulando tanto experiência vivida quanto conhecimento científico, para lidar com o processo de morte encefálica e doação de órgãos e tecidos. Ser enfermeiro atuante nesse processo significa promover uma assistência de qualidade em todas as dimensões impactadas pela doação e transplante de órgãos, seja de forma direta ou indireta. É cuidar das pessoas envolvidas no processo, como o familiar, o potencial doador e o provável receptor de órgãos, oferecendo à sociedade um cuidado de enfermagem digno, ético e humanístico.
